# The storm inside: Abdominal and urinary complications in lupus

**DOI:** 10.1515/rir-2025-0024

**Published:** 2025-10-04

**Authors:** Shaoyu Zheng, Guangzhou Du, Yukai Wang

**Affiliations:** Department of Rheumatology and Immunology, Shantou Central Hospital, Shantou, Guangdong Province, China; Department of Radiology, Shantou Central Hospital, Shantou, Guangdong Province, China

**Figure 1:**
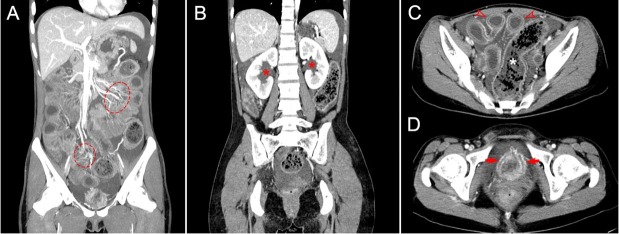
Computed tomography (CT) imaging features of lupus enteritis and cystitis. A: Coronal CT image shows mesenteric vessel engorgement, manifesting as the “comb sign”(dotted circle), a characteristic finding of mesenteric inflammation; B: Coronal CT image reveals renal pelvic dilation (red asterisk), indicative of hydronephrosis, with findings raising concern for urinary tract involvement; C: Transverse CT image demonstrates small bowel wall thickening exhibiting the “target sign”(hollow red arrow), accompanied by luminal dilation, intraluminal fluid accumulation, and ascites. Additionally, there is marked colonic and sigmoid dilation with associated wall thinning (white asterisk); D: Transverse pelvic CT image shows bladder wall thickening with edema (red arrow), characteristic of lupus cystitis.

A 25-year-old woman presented with a 2-year history of abdominal pain, distension, and loose stools 3–4 times daily. She also reported nausea, vomiting, and frequent urination (20–30 times/day) without dysuria. She denied fever, rash, oral ulcers, dry eyes and dry mouth, joint pain, or Raynaud’s phenomenon. Initial diagnoses of acute gastroenteritis and gastrointestinal infection led to proton pump inhibitor and antibiotic therapy, but her symptoms recurred persistently. Physical examination revealed reduced bowel sounds and diffuse abdominal tenderness without rebound tenderness. Murphy’s sign was negative. Laboratory findings included positive antinuclear antibodies with a titer of 1: 1000, anti-double-stranded DNA auto-antibodies (65, normal range < 9), and anti-Sjogren syndrome A antibody (anti-SSA) antibodies (45, normal range < 9). She also had leukopenia (white blood cell count: 2.0×109/L, normal range 4.0–10.0×109/L) and hypocomplementemia (C3: 0.62 g/L, normal range 0.79–1.52 g/L; C4: 0.08 g/L, normal range 0.16–0.38 g/L). D-dimer was markedly elevated (33, 200 μg/L, normal range < 550 μg/L). Urinalysis showed proteinuria (+++) without leukocytes, and 24-hour urinary protein excretion was 1.147 g. Antiphospholipid antibodies, antineutrophil cytoplasmic antibody, C-reactive protein, procalcitonin, erythrocyte sendimentation rate, and tumor markers were unremarkable. Abdominal computed tomography (CT) scanning (Figure 1A, 1C) revealed extensive gastric and small bowel wall thickening, an obvious “target” sign due to submucosal edema, a “comb” sign due to increased number of mesenteric vessels, luminal dilation, fluid accumulation, and ascites. Hydronephrosis (Figure 1B) and bladder wall thickening (Figure 1D) were also noted. Endoscopy confirmed chronic gastritis and intestinal mucosal hyperemia. There was no evidence of tumor or infection. The patient was eventually diagnosed with systemic lupus erythematosus (SLE) manifesting as lupus enteritis (LE), nephritis, and cystitis. After two weeks of treatment with high-dose glucocorticoids (2 mg/kg), cyclophosphamide (500 mg per two weeks), belimumab (10 mg/kg), hydroxychloroquine, and anticoagulant agent, the patient’s condition has shown significant improvement.

Lupus enteritis account for 45% of acute abdominal pain in SLE, and often coexisting with bladder and ureteral involvement.^[1,2]^ The three most common features includes the target sign, comb sign, and increased attenuation of mesenteric fat.^[3]^ Intestinal pseudo-obstruction is highly suspected when abdominal CT shows dilated bowel loops, air-fluid levels, wall thickening, or edema, but absence of a mechanical cause.^[4]^ Although abdominal CT imaging is sensitive, these findings are nonspecific and can also be seen with other conditions, such as intestinal obstruction, pancreatitis, or inflammatory bowel disease).^[1]^ Therefore, accurate diagnosis of abdominal and urinary tract involvement in SLE necessitates a comprehensive synthesis of clinical evaluation, serological testing, and imaging findings to delineate disease extent and distinguish SLE-related pathology from alternative etiologies.
